# Physical Basis of Multi-Energy Coupling-Driven Water Oxidation

**DOI:** 10.3389/fchem.2022.902814

**Published:** 2022-05-09

**Authors:** Zijiao Han, Shun Yuan, Duanduan Liu, Qian Zheng, Yu An Huang, Shicheng Yan, Zhigang Zou

**Affiliations:** ^1^ Shenyang University of Technology, Shenyang, China; ^2^ State Grid Liaoning Electric Power Supply Co. Ltd., Shenyang, China; ^3^ Northeast China Energy Regulatory Bureau of National Energy Administration, Shenyang, China; ^4^ Jiangsu Key Laboratory for Nano Technology, Eco-materials and Renewable Energy Research Center (ERERC), College of Engineering and Applied Sciences, Nanjing University, Nanjing, China; ^5^ Department of Physics, Nanjing University, Nanjing, China; ^6^ School of Materials Science and Engineering, Nanjing Institute of Technology, Nanjing, China

**Keywords:** water oxidation, multi-energy coupling, hydrogen production, electrocatalysis, heat

## Abstract

Hydrogen production by electrolyzing water is an important technique to store energy from renewables into chemical energy. Many efforts have been made to improve the energy conversion efficiency. In this review article, we mainly summarized the emerging ideas on water oxidation by multi-energy coupling. First, the physicochemical nature of electrolyzing water reaction is described. Then, we conceptually proposed the physical basis of energy coupling with a goal to maximize the energy conversion efficiency and showed the methods to achieve heat–electricity and magnetism–electricity coupling to drive water splitting. Finally, the material requirements for creating efficient energy coupling water splitting system were proposed. These new ideas unlock a big potential direction for developing multi-energy coupling hydrogen production devices to efficiently store the intermittent and fluctuating renewables.

## Introduction

Today, we are living in a great historic era of energy revolution from the carbon cycle to the hydrogen cycle all around the world (Jacobson et al., 2017). To build a stable and efficient renewable supply system, hydrogen is the ideal medium for long-term storage and long-range transport of intermittent and unevenly distributed renewables. A feasible route is to produce hydrogen by temporary excess electricity electrolyzing water. A key challenge is to maximize the energy conversion efficiency from renewables to chemical energy. The kinetically sluggish oxygen evolution reaction (OER) is the rate-determining step in various energy processes, such as solar- or electricity-driven water splitting (2H_2_O → 2H_2_ + O_2_) (Suntivich et al., 2011; Landman et al., 2017), CO_2_ fixation (x CO_2_ + *y*/2 H_2_O → C_x_H_y_O_z_ + (4x + y-z)/4 O_2_) (Asadi et al., 2016), and rechargeable metal–air batteries (M_x_O → xM + 1/2 O_2_) (Peng et al., 2020). Many efforts have been made to accelerate the OER kinetics. The previous achievements were focused on adjusting the electronic structure of the catalyst to decrease OER barriers and finding the effective physical parameters as the OER descriptor to guide the design of efficient catalysts (Song et al., 2020). In addition to developing catalytic materials, the multi-energy coupling is another route to enlarge the energy conversion efficiency. In this article, we summarized the basic principles, challenges, and recent advances for promoting OER kinetics by multi-energy coupling.

## Thermodynamics of Water Splitting

In principle, water splitting is an endothermic reaction. We can express the water splitting reaction under standard conditions as follows: H_2_O+ 237.2 kJ/mol electricity +48.6 kJ/mol heat → H_2_ + ½ O_2_, where 237.2 kJ/mol is the free energy of formation of water, ∆G_f_, and 48.6 kJ/mol is the irreversible heat of chemical reaction, T∆S (T, absolute temperature; ∆S, entropy change). Accordingly, the potential requirement for water electrolysis is 1.229 V at 25 C (E^o^_H2O_ = ∆G_f_/2F, Faraday constant, 96,485 C mol^−1^), assuming that the external heat is supplied. If all the energy requirement of water splitting is supplied from electricity, the thermoneutral potential for water electrolysis, E_tneut_, describing the system state at enthalpy balance, is 1.481 V [E_tneut_ = ∆H/2F = (∆G_f_ + T∆S)/2F, ∆H, enthalpy change] (Licht, 2003). At this voltage, the produced heat from internal resistance for the electric and ionic currents flowing through the cell and the irreversible heat of chemical reaction provide an exact feedback to the heat requirement for water splitting. The cell operating voltage above 1.481 V will produce excess heat. Therefore, the main task of water electrolysis is to decrease the irreversible heat of the OER.

**FIGURE 1:**
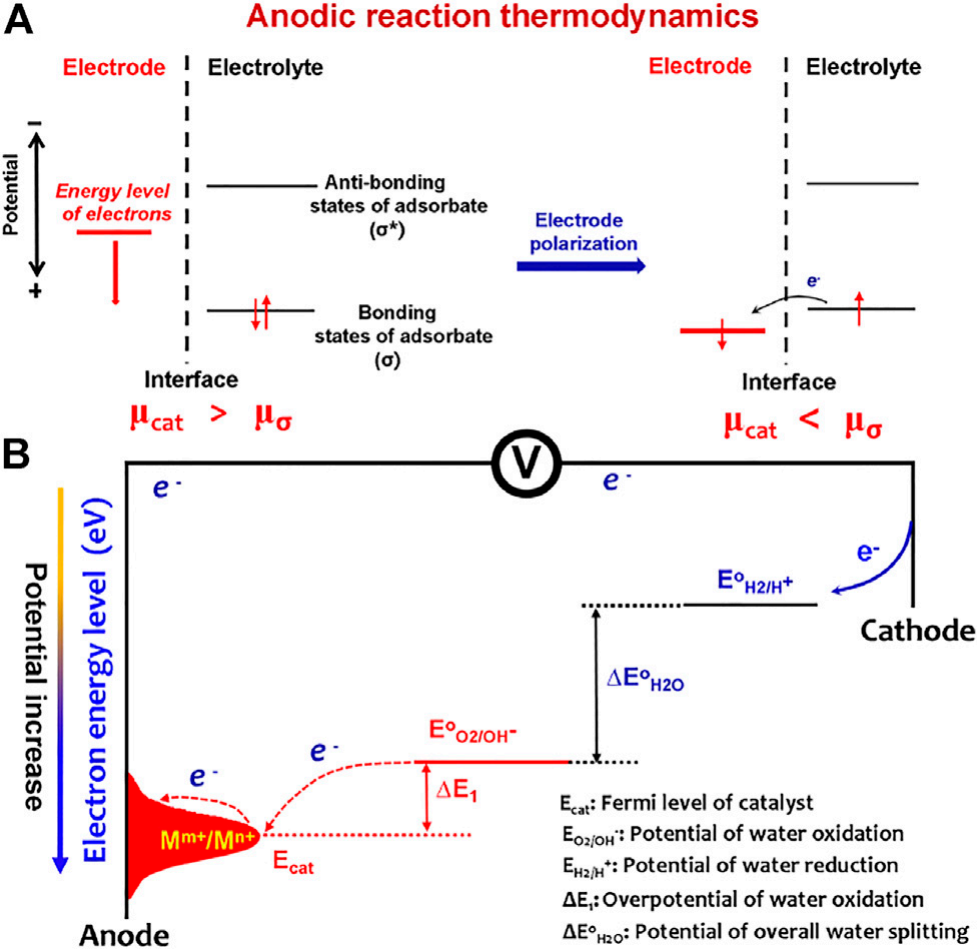
**(A)** Anodic reaction thermodynamics. After electrode–electrolyte contact, the initial equilibrium state between the electrode catalyst and OER adsorbates was established by orbital hybridization interactions. When applying external potentials, the Fermi level (*μ*_cat_) of catalyst is polarized to an energy level lower than the bonding states (*μ*_σ_) of adsorbates; thus, the OER process occurs. **(B)** Barriers of the OER to occur on an electrode include the barriers from electrode catalyst polarization by varying valency of ions (M^m+^/M^n+^) and the barriers from water oxidation by high-valence ions (M^n+^).

From the view of molecular and electronic aspects of the interface reaction, it has been well recognized that the OER is mainly driven by the atom-scale interactions between the catalytic materials and OER intermediates (^*^OH, ^*^O, and ^*^OOH) (Bajdich et al., 2013). The catalysts function via the orbital interactions with OER intermediates to form bonding (*σ*) and antibonding (*σ* ^*^) states (Figure 1A). After electrode–electrolyte contact, the initial equilibrium state between the electrode catalyst and OER adsorbates was established by orbital hybridization interactions. At this situation, the Fermi levels of the catalyst and OER adsorbates are equal, and the Fermi level (*μ*_cat_) of catalyst is higher than the bonding states of adsorbates. When applying external potentials, *μ*_cat_ is polarized to an energy level lower than the bonding states (*μ*_*σ*_) of adsorbates; thus, the OER process occurs. Most catalysts follow the solid-state redox couple mechanism (Liu et al., 2021), that is, the variable-valency ions (M^m+^/M^n+^) of catalysts are the charge transfer medium to decrease OER barriers (Figure 1B). The applied potentials increase the average valence states of the variable-valency ions, thus downshifting the Fermi level (E_cat_) of the catalyst. Accordingly, the OER barriers would originate from two electron transfer processes: the electron transfer for redox couple oxidation to form high-valence ions (electrode polarization process) and high-valence ions extracting electrons from OER intermediates (water oxidation process). This means that minimizing the barriers of the two electron transfer processes is an effective route to accelerate water splitting.

**FIGURE 2:**
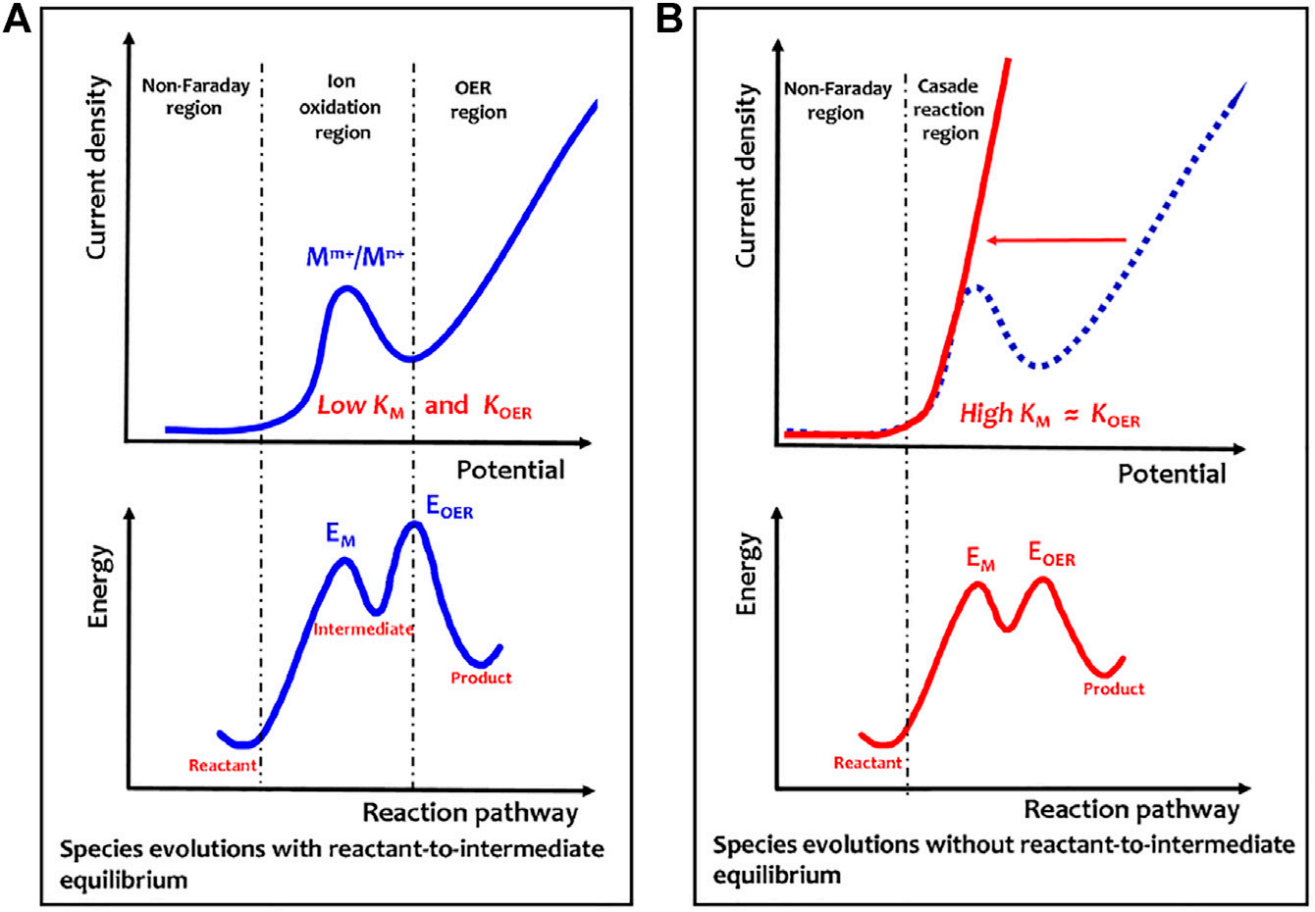
Electricity–heat coupling mechanism of electrochemical–chemical cascaded water oxidation. **(A)** Anodic polarization curve of room temperature electrochemical–chemical two-step water oxidation. **(B)** Anodic polarization curve of heat-triggered electrochemical–chemical cascaded water oxidation. The energy profile is shown here to describe energy barriers for different reaction coordinates. Evidently, in Figure 2A, much higher barrier energy for E_OER_ than E_M_ will induce a possible situation to establish a complete equilibrium between the reactant and intermediate. However, in Figure 2B, the two energy barriers for E_OER_ and E_M_ are nearly equal, so no complete equilibrium is established between the reactant and intermediate, thus creating a nearly steady-state cascade reaction from the reactant to product. Reprinted with permission from Ref.12. Copyright ^©^ 2022, American Chemical Society.

Evidently, for the solid-state redox couple-mediated water splitting, electricity is in charge of polarizing the catalyst to a given energy level that allows spontaneous chemical water oxidation to occur. As described by the energy profile shown in Figure 2, the overpotentials involve both M^m+^/M^n+^ oxidation (barrier, *E*_*M*_; rate constant, *K*_*M*_) and polarizing M^n+^ species to trigger chemical water oxidation (barrier, *E*_*OER*_; rate constant, *K*_*OER*_). The difference between *E*_*OER*_ and *E*_*M*_ determines the kinetic reaction route. When the *E*_*OER*_ is much higher than *E*_*M*_, the higher difficulty for the OER to occur induces a possibility to establish a complete equilibrium between M^m+^/M^n+^ oxidation and the proton reduction to the product hydrogen (Figure 2A). Indeed, the oxidation potential gap (*∆*V) between Ni^2+^/Ni^3+^ and OER is generally large enough to create a hydrogen generation reaction by an anodic oxidation of Ni(OH)_2_ to NiOOH and a cathodic reduction of H_2_O to H_2_ (Chen et al., 2016; Dotan et al., 2019). However, if the *E*_*M*_ and *E*_*OER*_ barriers are nearly equal (energy profile in Figure 1B), there is no complete equilibrium between reactants and intermediates, and a nearly steady-state cascade reaction from reactants to products is created. In this situation, a highly efficient OER process is expected to accelerate the *K*_*M*_ and *K*_*OER*_ to a nearly same rate at the initial oxidation potential of redox couple without kinetic delay, thus achieving a consecutive two-step cascade reaction (Liu et al., 2022a).

## The Physical Basis of Energy Coupling

**FIGURE 3:**
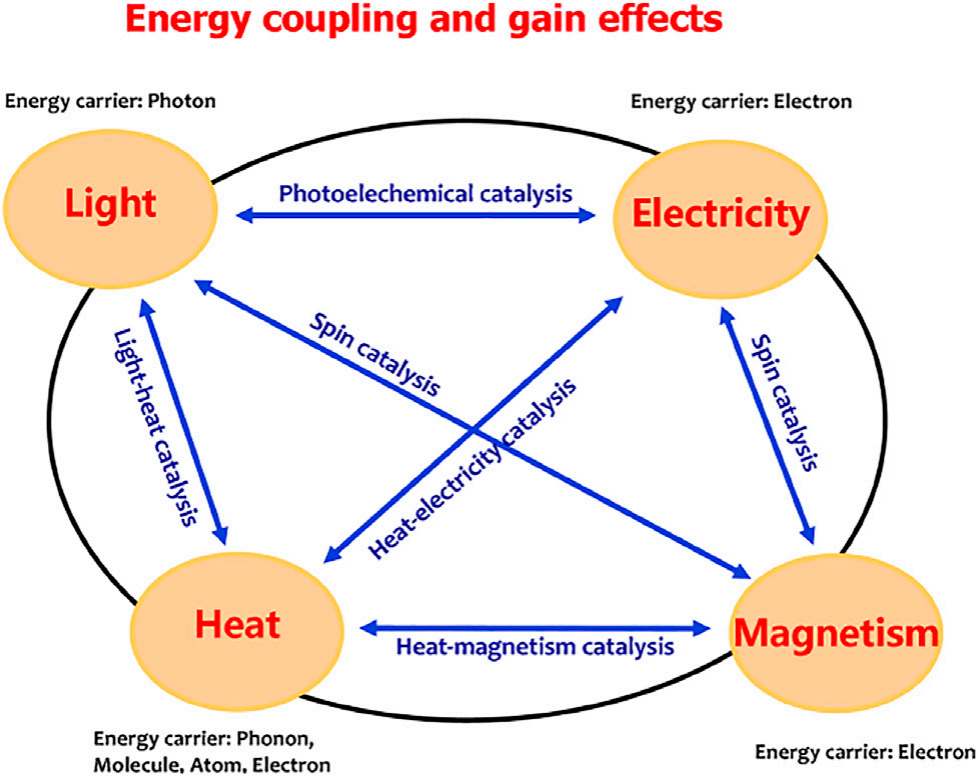
Coupling and gain effects between two common energies.

As described in Figure 3, light, heat, electricity, and magnetism are the most common forms of energy. The energy carrier is the photons for light energy, the electrons for electricity and magnetism energies, and the phonons, molecules, atoms, or electrons for heat energy. Many efforts have been made to achieve efficient energy coupling. Various energy coupling systems, such as the light coupling with electricity to create photoelectrochemical catalysis (Fujishima and Honda, 1972), magnetism coupling with light or electricity to achieve spin catalysis, and the heat–electricity coupling catalysis (Mtangi et al., 2015; Garcés-Pineda et al., 2019; Ren et al., 2021; Yan et al., 2021), were developed to drive water splitting. In these systems, the required electrical energy input was partially replaced by light, magnetism, or thermal energy. Maximizing the energy efficiency is a significant challenge in the case of multi-energy inputs, depending on the discovery of energy coupling mechanism. The photoelectrochemical catalysis is the well-known light–electricity coupling water splitting based on a semiconductor–liquid junction mechanism (Song et al., 2022). In this system, the external electric field contributes to promoting photogenerated charge separation and extraction and the polarization of the catalytic layer to meet energy-level requirement of water oxidation. Therefore, the external electric field changes the charge concentration and the electron chemical potential, the process which makes the light–electricity coupling efficient. For an electrochemical reaction, energy conversion to occur is driven by the chemical potential of electrons, that is, the electrons are the energy carriers; thus, the chemical potential gradient of electrons is a direct criterion to show the energy conversion route. Therefore, an efficient energy coupling process would indicate that the energies in a system act together to change the electronic chemical potential of the system.

## Advances in Multi-Energy Coupling Water Splitting

### Magnetism–Electricity Coupling Water Splitting

**FIGURE 4:**
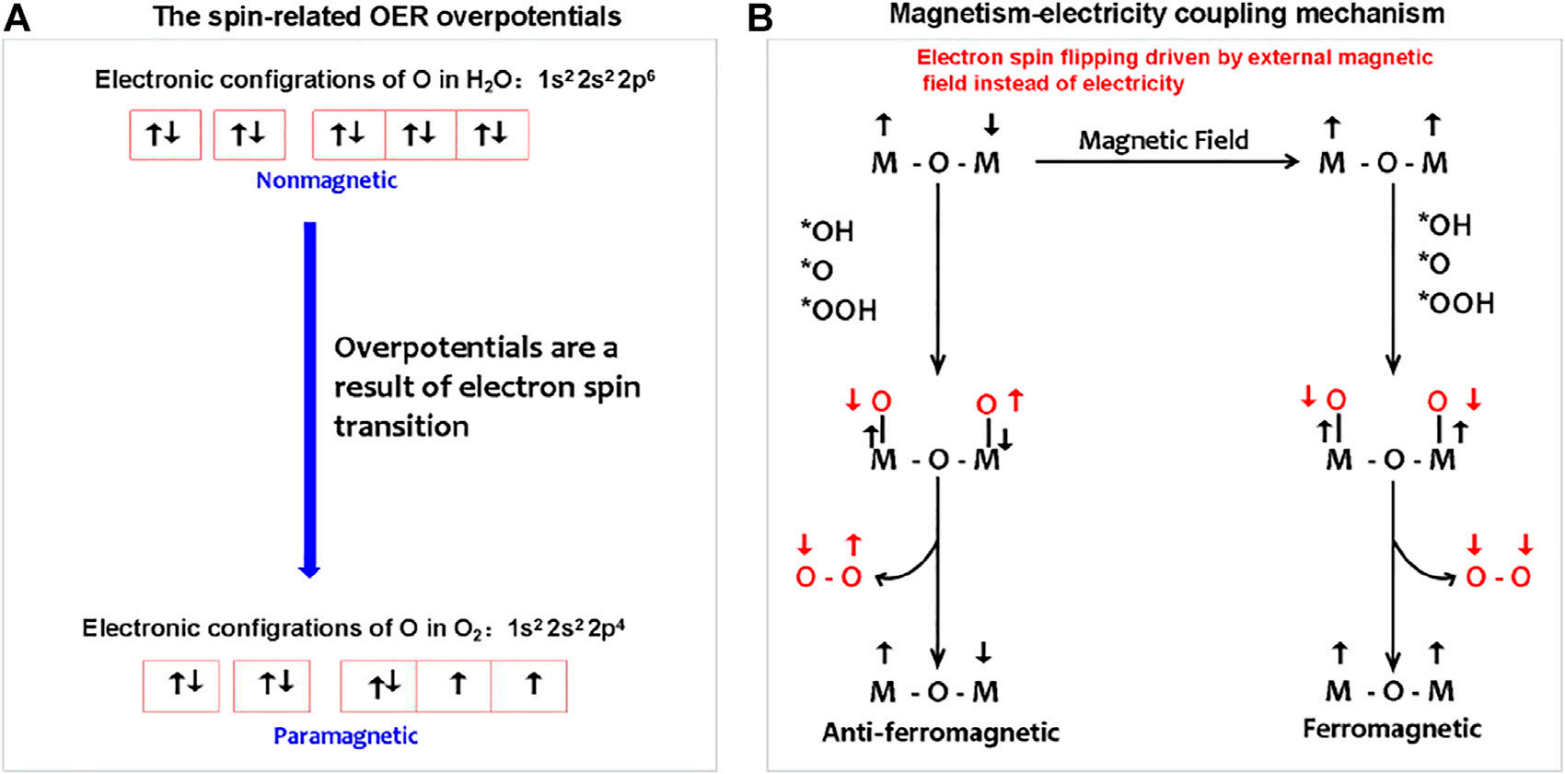
**(A)** Spin-related OER overpotentials from electron spin changes during nonmagnetic water splitting to paramagnetic O_2_. **(B)** Magnetism–electricity coupling water splitting follows the mechanism of magnetic field, instead of electricity-driven spin ordering. The magnetic field-driven magnetic transition from anti-ferromagnetic to ferromagnetic is adopted as an example to describe the magnetism–electricity coupling mechanism.

As shown in Figure 4, for the OER, with regard to magnetic electronics, the paramagnetic triplet state O_2_ product with two unpaired electrons generated from the initial nonmagnetic singlet bound state OH^−^/H_2_O reactants without unpaired electrons (Li et al., 2021). The spin-state change driven by electric polarization is necessary to achieve the proton-coupled spin-related electron transfer (PCSRET) during water oxidation and probably is the main origination of redox couple-mediated OER overpotentials. In theory, a magnetic anode may catalyze the paramagnetic oxygen release from nonmagnetic water in ground state, which process is lower barrier compared to that the oxygen on the nonmagnetic anode can be generated only in an excited nonmagnetic state without violating the spin conservation rule. In addition, the magnetic electrode is able to respond to the external magnetic field to achieve the magnetism–electricity coupling. Recently, Prof. Xu’s group has explored the spin-dependent kinetics *via* exchange interactions, spin–orbit coupling, or spin pinning effect to catalyze OER electrochemistry (Sun et al., 2020; Chen et al., 2021; Ren et al., 2021; Sun et al., 2021; Wu et al., 2021). The efforts on magnetism–electricity coupling were mainly focused on two research directions: finding the materials with strong magnetic response and creating magnetic interface interactions to induce spin ordering of the catalytic layer.

The magnetism originates from the spin and orbital magnetic moment of an electron. The external magnetic field imposing on magnetic materials may induce the splitting of energy levels and the changing of spin–orbit coupling, that is, the interaction of an electron’s spin magnetic moment with its orbital magnetic moment. This means that the external magnetic field can change the energy state of electrons, thus contributing to the chemical potential change of electrons, that is, the external magnetic field is an important energy to maintain the energy state of electrons.

The magnetism–electricity coupling requires materials with high sensitivity to magnetic field. This means that the materials should contain the magnetic OER-active elements, which largely limits the amount of available elements for designing the catalytic materials. Meanwhile, the visible magnetism–electricity coupling effect was observed under a high-intensity magnetic field, resulting from the low magnetic sensitivity of the catalytic materials. The use of high-intensity magnetic field increases the difficulty for large-scale applications of magnetism–electricity coupling water splitting technology. In addition, under an electrolyte environment, the surface amorphization of catalysts is a common phenomenon due to the electrochemical corrosion. The amorphous structure usually has a weak response to the external magnetic field, originating from the frustrated exchange interaction in short-range ordered structures. The inevitable surface amorphization inducing weak magnetic response may be compensated by the interface interactions from a strong magnetic core.

### Heat–Electricity Coupling Water Splitting

**FIGURE 5:**
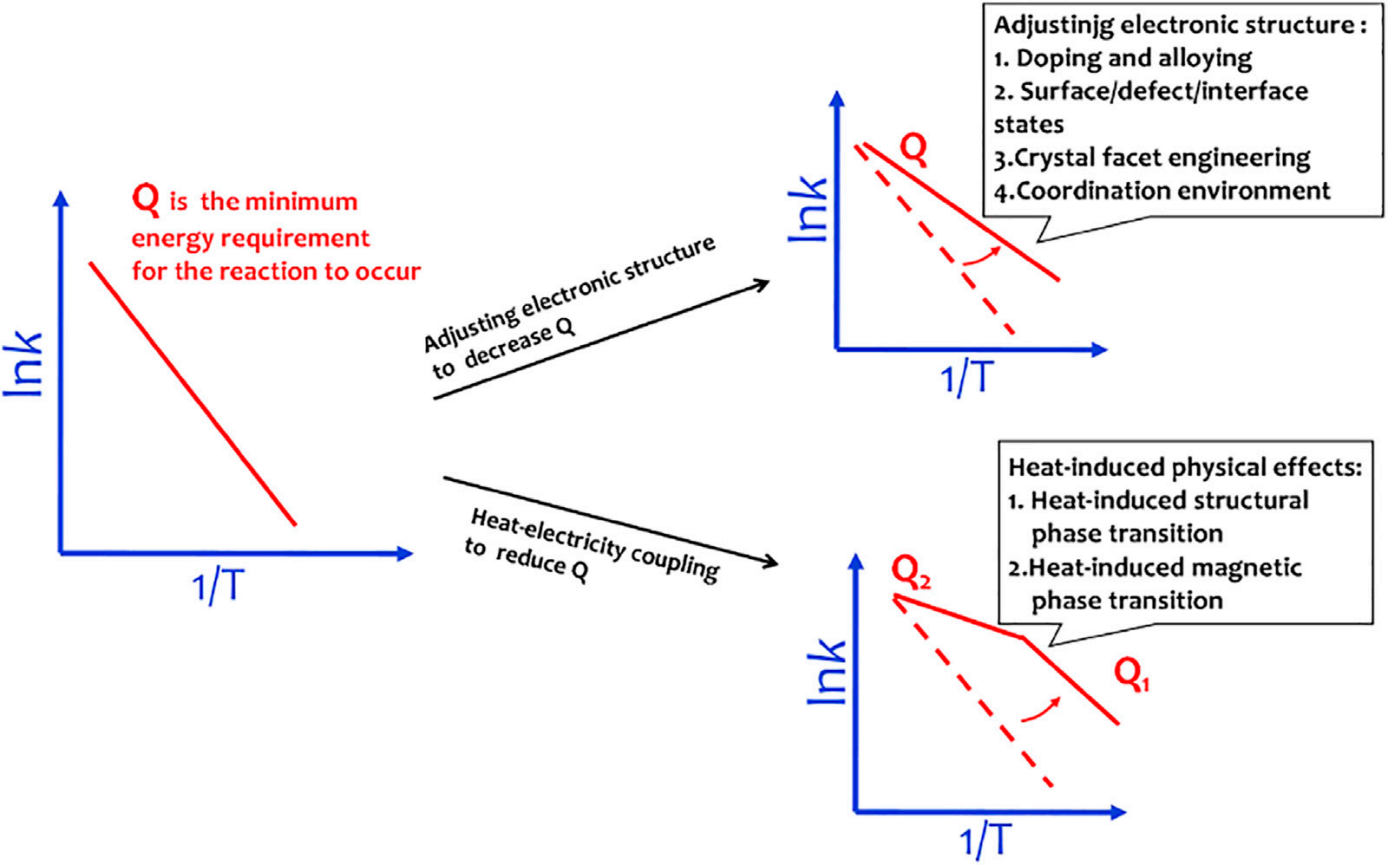
Materials designed by adjusting electronic structures to decrease activation energy follow the linear Arrhenius relationship. The materials with heat-induced physical effects can induce a sudden change in activation energy.

In principle, the endothermic water splitting reaction needs simultaneous input of electricity and heat. Although heat as an important energy is widely produced *via* industrial, solar–thermal, or geothermal processes, the heat–electricity coupling is only well known by the Arrhenius relationship that describes the dependence of the rate constant of an electrochemical reaction on the absolute temperature (Laidler, 1984). The activation energy (Q) can be calculated by a linear relationship between ln*k* and T^−1^ from the Arrhenius relationship, ln*k* = −Q/(RT) + ln*A*, where *k* is the rate constant, A is the pre-exponential factor, R is the universal gas constant, and T is the absolute temperature. It is well accepted that the heat coupling to the electrochemical process contributes to improvement of the mass transport (Ganley, 2009). The materials designed by adjusting electronic structures are able to efficiently decrease OER activation energy. To obey the Sabatier principle, a good OER site should adsorb ^*^OOH weaker than ^*^OH for easy initial reactant capture and final product desorption. Adjusting the electronic structures of the catalyst will change the interactions between OER intermediates and catalysts, thus well matching the Sabatier principle. However, when these materials work at 50–80°C in a real electrolyzer, the water splitting rate well follows the linear relationship between ln*k* and T^−1^ with elevation in the temperature of the system. This means that in this situation, heat only accelerates the mass transport of water splitting reaction and does not contribute to the changes of electronic chemical potential, that is, the materials designed by adjusting electronic structures are usually unable to achieve the efficient heat–electricity coupling in the operating temperature region of the electrolyzer (Figure 5).

Recently, Yan’s group edited [Ni_3_OH_2_(H_2_O)_4_]_n_^4n+^ chains stocked Ni BDC metal–organic frameworks (MOFs) by a chemical etching–assisted electrooxidation to produce *γ*-NiOOH with intercalated Ni-O species (Liu et al., 2022a). The Ni–O intercalations with high structural degrees of freedom are highly sensitive to heat and electricity. In addition, the Ni–O intercalations stabilize the low-barrier interconversion of *α*-Ni(OH)_2_/*γ*-NiOOH redox couple, allowing electricity–heat complementary water splitting. More importantly, in this route, the Ni–O intercalations can directly trigger the thermochemical reaction of electricity-polarized high-valence Ni ions with H_2_O to generate O_2_, significantly lowering the barrier energies of initial electrooxidation of Ni^2+^/Ni^3+^ and subsequent chemical water oxidation to the nearly equal value via coupling a low-grade heat field (<100°C), thereby achieving a consecutive two-step cascade reaction without kinetic delay. Heat is able to trigger the electron transition from O 2p orbitals of OER intermediates into the valence orbitals of high-valency redox couple when the external electric field electrochemically polarized the redox couple to reach a given energy level. Therefore, in principle, the heat exciting electron transition can partially replace electricity and contribute to the OER to occur. This work demonstrated a possibility to cascade the electrochemical and thermochemical reactions, thus achieving the heat–electricity coupling.

Figure 2Bshows that redox couple oxidation as an initial step of water oxidation may be key for high electricity consumption in the electrochemical OER. If the energy input can change the electronic states of catalysts, the redox couple oxidation kinetics may be accelerated. The heat-induced magnetic phase transformation or heat-induced structural phase transformation has well-known heat-induced physical effects. Recently, we have demonstrated that the heat-induced magnetic transition is an efficient strategy to speed up the oxidation kinetics of redox couples (Liu et al., 2022b). The activation energy of Ni^2+^/Ni^3+^ redox couple oxidation was sharply decreased by heating the Ni_0.67_Fe_0.33_O_x_H_y_ catalyst above a Curie temperature (T_c_) of 70°C. In such a strategy, heat, instead of electricity, drives the spin flipping of Ni^2+^/Ni^3+^ oxidation through heat-sensitive ferri-to-paramagnetic spin-state changes. As a result of magnetic transition-assisted efficient heat–electricity coupling, Ni_0.67_Fe_0.33_O_x_H_y_ exhibits the lowest OER overpotential of 221 mV at 100 mA cm^−2^ at 90°C in alkaline electrolytes, outperforming the benchmark IrO_2_ catalyst. This work provides new insights into the design of efficient heat–electricity complementary OER devices.

## Summary

Light, heat, and wind are important renewables. To convert light and wind to electricity and subsequently store the electricity as chemical energy are a route to build a stable renewable supply 100% of world’s power. The electricity–heat coupling is a common and vital energy coupling route. The heat-induced physical effects provide a new idea to design materials that can highly respond to heat and electricity to change the chemical potential of electrons for low-barrier electrode polarization and electron transfer during water splitting. The designing ideas in highly efficient heat–electricity coupling based on heat-induced physical effects help us better understand more energy coupling routes.

1) Potential multi-energy coupling routes for efficient water splitting.

**FIGURE 6:**
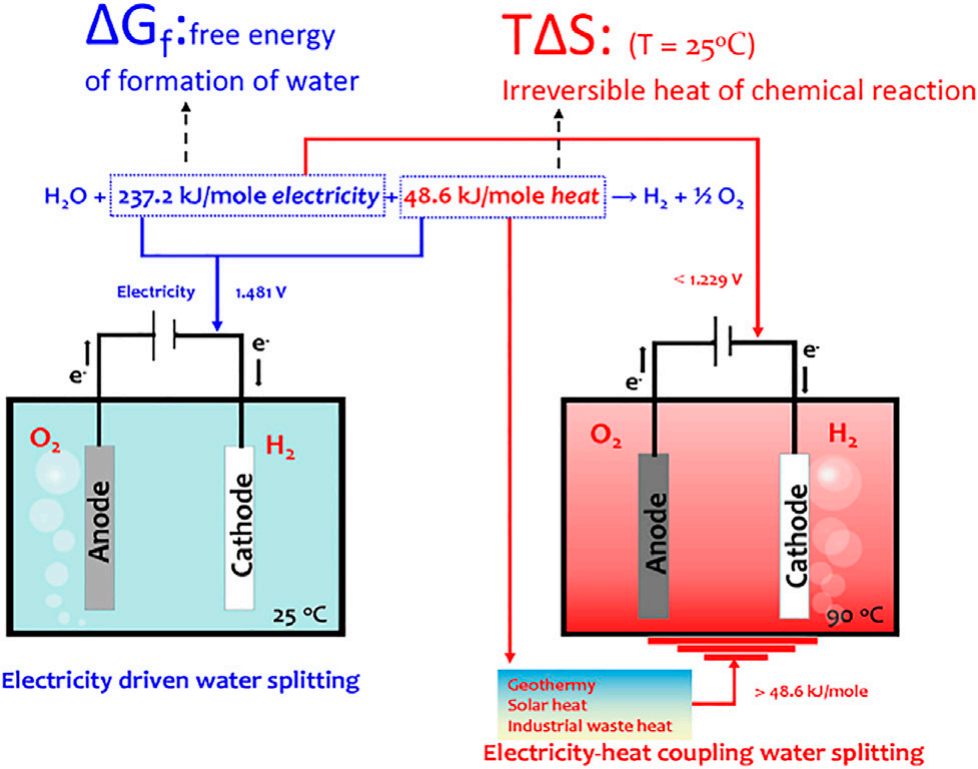
Heat–electricity coupling electrolyzer to show the revolution in device designing guided by multi-energy coupling ideas. Reprinted with permission from Ref.12. Copyright ^©^ 2022, American Chemical Society.

As shown in Figure 6, to discover the physical basis of heat coupling with other energies is very important because there are abundant sources of heat in nature, and utilization and conversion of various energies accompany heat generation. In particular, for storing renewables by electrochemical hydrogen production, the heat–electricity coupling is the most important energy coupling. Light can be conveniently converted to heat by concentrating solar- or light-induced plasmon resonance effect. Specifically, the light-induced plasmon resonance effect of metal particles can produce heat and induce a strong electrostatic field. The plasmon resonance effect can achieve the local heating and electrostatic field effect to contribute to the chemical potential changes of electrons; thus, it is a potential effective method to create light–electricity–heat coupling water splitting *via* an electrocatalytic or photoelectrocatalytic route. In addition, the electrons of a shallow donor state are able to be excited by heat into conduction band; thus, creating surface states is a potential route to achieve heat–electricity–heat photoelectrochemical water splitting. In this route, the shallow donor states are an important electron transfer station for capturing and storing photogenerated electrons and heat takes part in the electron extraction from shallow donor states into conduction band, thus partially instead of electricity contributing to the charge separation. The abundant heat-induced physical effects, such as heat-induced magnetic or structural phase transition, pyroelectricity, heat excitation of charges, and heat stress, provide more possibilities to achieve highly efficient energy coupling.

2) Challenges in multi-energy coupling devices.

The new energy coupling mechanism would lead to the revolution of device design, such as the heat–electricity coupling hydrogen production system comprising of concentrating solar heating the electrolyzer, which can operate at heating with fast start-up speed and less electricity input. To achieve such multi-energy coupling system, innovations in miniaturization of high-performance devices, energy management system, and system integration technology are required. Energy coupling would increase the complexity of the advanced energy conversion system; thus, it is needed to develop a device miniaturization technique for convenient large-scale applications, develop an energy management system with an energy optimization constraint by heat-induced physical effects, and develop system integration technology to decrease energy loss.

All in all, to build the multi-energy coupling system, we need to further explore the physical basis of energy coupling. The advantages of multi-energy coupling meet the requirement of high-energy efficiency of the system; thus, it would be the vital striving direction for new energy source industry.
